# Frequent Infection of Cats With SARS-CoV-2 Irrespective of Pre-Existing Enzootic Coronavirus Immunity, Brazil 2020

**DOI:** 10.3389/fimmu.2022.857322

**Published:** 2022-04-05

**Authors:** Edmilson F. de Oliveira-Filho, Otávio V. de Carvalho, Ianei O. Carneiro, Fagner D’ambroso Fernandes, Sara Nunes Vaz, Célia Pedroso, Lilian Gonzalez-Auza, Victor Carvalho Urbieta, Arne Kühne, Rafaela Mayoral, Wendy K. Jo, Andrés Moreira-Soto, Chantal B. E. M. Reusken, Christian Drosten, Carlos Brites, Klaus Osterrieder, Eduardo Martins Netto, Luiz Eduardo Ristow, Rita de Cassia Maia, Fernanda S. Flores Vogel, Nadia Rossi de Almeida, Carlos Roberto Franke, Jan Felix Drexler

**Affiliations:** ^1^ Institute of Virology, Charité-Universitätsmedizin Berlin, Corporate Member of Freie Universität Berlin, Humboldt-Universität zu Berlin, Berlin, Germany; ^2^ Tecsa Laboratorios, Belo Horizonte, Brazil; ^3^ School of Veterinary Medicine and Zootechny, Federal University of Bahia, Salvador, Brazil; ^4^ Department of Veterinary Medicine, Federal University of Santa Maria, Santa Maria, Brazil; ^5^ Disease Research Laboratory, University Hospital Professor Edgard Santos, Federal University of Bahia, Salvador, Brazil; ^6^ Centre for Infectious Disease Control, National Institute for Public Health and the Environment (RIVM), Bilthoven, Netherlands; ^7^ Institut für Virologie, Freie Universität Berlin, Berlin, Germany; ^8^ Veterinary Medicine Department, Federal Rural University of Pernambuco, Recife, Brazil; ^9^ German Centre for Infection Research (DZIF), Associated Partner Site Charité, Berlin, Germany

**Keywords:** SARS-CoV-2, cats (felis catus), serology, zoonosis, cross-reactivity, coronavirus

## Abstract

Carnivores such as cats and minks are highly susceptible to SARS-CoV-2. Brazil is a global COVID-19 hot spot and several cases of human-to-cat transmission have been documented. We investigated the spread of SARS-CoV-2 by testing 547 domestic cats sampled between July-November 2020 from seven states in southern, southeastern, and northeastern Brazil. Moreover, we investigated whether immune responses elicited by enzootic coronaviruses affect SARS-CoV-2 infection in cats. We found infection with significantly higher neutralizing antibody titers against the Gamma variant of concern, endemic in Brazil during 2020, than against an early SARS-CoV-2 B.1 isolate (p<0.0001), validating the use of Gamma for further testing. The overall SARS-CoV-2 seroprevalence in Brazilian cats during late 2020 validated by plaque reduction neutralization test (PRNT_90_) was 7.3% (95% CI, 5.3-9.8). There was no significant difference in SARS-CoV-2 seroprevalence in cats between Brazilian states, suggesting homogeneous infection levels ranging from 4.6% (95% CI, 2.2-8.4) to 11.4% (95% CI, 6.7-17.4; p=0.4438). Seroprevalence of the prototypic cat coronavirus Feline coronavirus (FCoV) in a PRNT_90_ was high at 33.3% (95% CI, 24.9-42.5) and seroprevalence of Bovine coronavirus (BCoV) was low at 1.7% (95% CI, 0.2-5.9) in a PRNT_90_. Neutralizing antibody titers were significantly lower for FCoV than for SARS-CoV-2 (p=0.0001), consistent with relatively more recent infection of cats with SARS-CoV-2. Neither the magnitude of SARS-CoV-2 antibody titers (p=0.6390), nor SARS-CoV-2 infection status were affected by FCoV serostatus (p=0.8863). Our data suggest that pre-existing immunity against enzootic coronaviruses neither prevents, nor enhances SARS-CoV-2 infection in cats. High SARS-CoV-2 seroprevalence already during the first year of the pandemic substantiates frequent infection of domestic cats and raises concerns on potential SARS-CoV-2 mutations escaping human immunity upon spillback.

## Introduction

SARS-CoV-2 has evolutionary origins in bats and emerged in humans during late 2019. Beyond humans, carnivores are particularly susceptible to SARS-CoV-2 (1). Among carnivores, natural SARS-CoV-2 infection has been shown in dogs, cats, lions, tigers, ferrets, and minks by late 2021 (2–7). Clinical presentation of SARS-CoV-2-infected cats includes respiratory and gastroenteric symptoms such as sneezing, coughing, nasal and ocular discharge, anorexia, vomiting, diarrhea and appetite loss, but COVID-19 in cats remains poorly described (4, 8). Molecular and serological evidence from several countries showed that cats can easily be infected with SARS-CoV-2, predominantly from infected humans, but also from minks and other cats (3, 5, 9, 10). During the 2003-2004 outbreak of SARS-CoV, it was speculated that mutations enabling efficient infection of humans may have arisen in carnivore intermediate hosts (1). In minks infected with SARS-CoV-2, mutations have arisen in a variant strain capable of infecting humans and showing reduced antibody-mediated neutralization by human serum samples (11–13).

Different from minks that are kept as farmed animals in some countries, over 370 million cats globally live in close contact with humans (see: https://www.statista.com/statistics/1044386/dog-and-cat-pet-population-worldwide/). Brazil, with roughly 24 million cats and 210 million inhabitants, is one of the countries most affected by the COVID-19 pandemic with high incidence, different variants of concern (VOC), and more than 600,000 deaths by the end of 2021 (14, 15). Household transmission from humans to cats has been reported in Brazil (16), but the epidemiology of SARS-CoV-2 infection in cats and its distribution over the country remain unknown.

Pre-existing coronavirus immunity may be protective against subsequent infection with SARS-CoV-2 (17, 18). In contrast, pre-existing immunity can also have deleterious effects, such as antibody-dependent enhancement (ADE) known to contribute to severe Dengue (19, 20). Among coronaviruses, ADE has been described during experimental Feline Coronavirus (FCoV) infection (21) and in consequence of vaccination with a recombinant vaccinia virus-based vaccine (22, 23). It is not known whether pre-existing immunity against enzootic carnivore coronaviruses would provide protection against or immune enhancement of SARS-CoV-2 infection. Additionally, previous infection by endemic human coronaviruses and other infection can cause false-positive results in SARS-CoV-2 antibody tests (18, 24–27), and careful assessments of coronavirus immunity are thus required irrespective of the host species. Here, we investigated SARS-CoV-2 seroprevalence in domestic cats from different Brazilian states and analyzed whether pre-existing coronavirus immunity affects SARS-CoV-2 diagnostics and infection.

## Materials and Methods

### Sampling

A total of 547 pandemic and 52 pre-pandemic serum samples from cats presenting miscellaneous clinical signs, including, among others, fever, weakness, lack of appetite, gastroenteritis, were sampled for routine laboratory diagnostics. Pandemic sera were sampled from July-November 2020 from seven states in Brazil (**Table 1**) and pre-pandemic sera were sampled in the states of Bahia and Rio Grande do Sul between May 2018 and November 2019. Based on the information available for 172 animals, the median age was 4 years, and the sex ratio (F/M) was 0.68 (70/102), suggesting our samples comprised mainly adult female animals. Sampling was approved by the Federal University of Bahia’s animal ethics committee under authorization no. 74/2019.

**Table 1:** SARS-CoV-2 seroprevalence in cats from different states in Brazil.

State	No. of samples	Seroprevalence % (95% CI)	Sampling period (2020)
Bahia	216	4.6 (2.2 – 8.4)	July-November
Minas Gerais	40	5.0 (0.6 – 16.9)	November
Pernambuco	23	8.7 (1.1 – 28.0)	September-November
Paraná	20	10.0 (1.2 – 31.7)	November
Rio de Janeiro	33	9.1 (1.9 – 24.3)	November
Rio Grande do Sul	57	8.8 (2.9 – 19.3)	November
São Paulo	158	11.4 (6.7 – 17.4)	November
Total	547	7.7 (5.3 – 9.8)	July-November

### Surrogate Virus Neutralization Test (sVNT)

For antibody screening we used a highly specific receptor-binding domain (RBD)-based SARS-CoV-2 surrogate virus neutralization test (sVNT) (cPass, GenScript, https://www.genscript.com), which does not require species-specific detection antibodies and should thus be usable in cats.

### Virus Neutralization Assay

**Figure 1:**
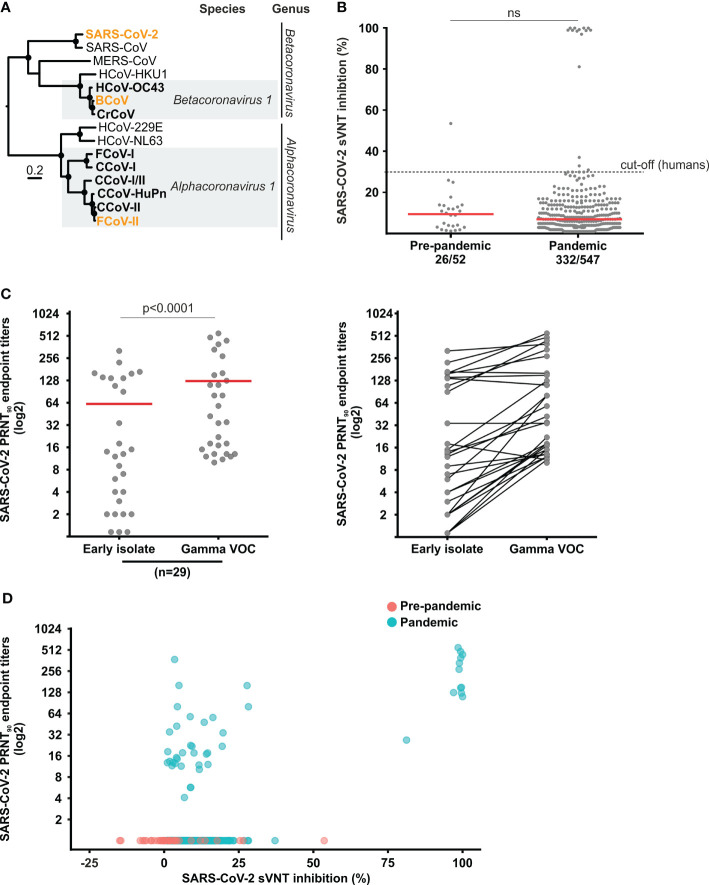
Coronavirus phylogeny and set-up of SARS-CoV-2 serology. **(A)** Maximum-likelihood tree based on translated spike gene sequences of human, dog and cat coronaviruses. The strains used for testing in this study are highlighted in orange. WAG+G+I was used as a substitution model and a complete deletion option was chosen. Scale bar indicates amino acid substitutions per site. Circles at nodes indicate support of grouping in ≥75% from 1,000 bootstrap replicates. **(B)** sVNT inhibition rate in sera from cats sampled before and during the pandemic. The 30% inhibition cut-off above which a human serum sample is considered positive is given for comparison. **(C)** Comparison of SARS-CoV-2 PRNT_90_ endpoint titers between an early isolate and a Gamma VOC strain. Statistical significance was determined using the Wilcoxon matched-pairs signed rank test. **(D)** Comparison of sVNT inhibition rates and reciprocal PRNT endpoint titers. Red bars in panels **(B, C)** indicate medians; ns, not statistically significant.

We performed plaque reduction neutralization tests (PRNT) and compared the reciprocal endpoint titers against SARS-CoV-2 with those against enzootic coronaviruses belonging to the viral species *Alphacoronavirus 1* and *Betacoronavirus 1* (**Figure 1A**). For SARS-CoV-2 we used the Gamma variant of concern (VOC) (strain hCoV-19/Netherlands/NoordHolland_10915/2021, purchased from the European Virus Archive Global) and an early B.1 isolate (Munich/ChVir929/2020 strain, sampled in January 2020; Gisaid accession: EPI_ISL_406862). For enzootic coronaviruses, we used FCoV (ATCC VR-989, WSU 79-1683 strain) and Bovine Coronavirus (BCoV) (strain Kakegawa) (**Figure 1A**). PRNT_90_ (a serum dilution reducing viral plaques by ≥90% is considered positive) was conducted in cell monolayers of 1.6 × 10^5^ Vero E6 for SARS-CoV-2, 3.5 × 10^5^ Crandell-Rees Feline Kidney cells (CRFK) for FCoV and 3.0 × 10^5^ sheep epithelioid cells (PT) (CCLV-RIE 0011) for BCoV. Cells were seeded in 12-well plates one day before the infection. Sixty plaque-forming units were incubated with serum dilutions of 1:20, 1:80, 1:320, and 1:1280 for one hour, added onto the cell monolayer, incubated again for one hour before adding the overlayer containing DMEM with 1% FCS and 2% Avicell for SARS-CoV-2 and 1.25% carboxymethyl cellulose (CMC) for FCoV and BCoV. After two days for SARS-CoV-2, four days for BCoV and five days for FCoV, the overlayer was removed, cells fixated with 6% paraformaldehyde and stained with crystal violet.

### Statistical Analysis

Endpoint reciprocal titers were calculated using a logistic regression function and statistical tests as indicated in the text and figure legends were done in GraphPad prism 6 (GraphPad Software, www.graphpad.com).

## Results

### Set-up of Serological Testing for SARS-CoV-2 Antibodies

To investigate whether pre-existing immune responses against enzootic coronaviruses or other pathogens affect SARS-CoV-2 antibody testing, we tested 52 pre-pandemic sera in a the receptor-binding domain (RBD)-based SARS-CoV-2 surrogate virus neutralization test (sVNT) considered highly specific for human antibody responses (28). Surprisingly, one cat sample (1.9%, 95% CI, 0.1-10.6) was clearly positive (signal inhibition ≥30.0%) and several other samples showed reactivity close to the sVNT threshold above which test results are considered positive in human-derived samples (**Figure 1B**). This suggested that sVNT results needed to be confirmed by PRNT. Because the emergence of the Gamma VOC in Brazil between June-November 2020 (14) overlaps with our sampling, we compared the PRNT_90_ endpoint titers against the Gamma VOC and an early SARS-CoV-2 isolate in a subset of PRNT-positive serum samples with sufficient volume. Our data confirmed significantly higher PRNT_90_ endpoint titers for the Gamma VOC in 24 out of 29 (82.8%) positive samples (Wilcoxon matched-pairs signed rank test, p<0.0001) (**Figure 1C**), which led us to conduct subsequent confirmatory testing only with the Gamma VOC strain. Because of relatively lower sVNT sensitivity due to usage of a spike protein RBD from an early isolate that may afford decreased reactivity with antibodies raised by contemporary strains in Brazil in the sVNT, all reactive sera (signal inhibition ratio ≥1.0) were confirmed by plaque reduction neutralization test (PRNT_90_) using the Gamma VOC, instead of using the 30% threshold used for testing of humans. Lastly, none of the pre-pandemic sera showing reactivity in the sVNT could be confirmed in the SARS-CoV-2 PRNT_90_, highlighting the risk of false-positive test results even when using a test validated for confirmatory human testing in cats (**Figure 1D**).

### Screening of Sera Sampled During the Pandemic for SARS-CoV-2 Antibodies

**Figure 2:**
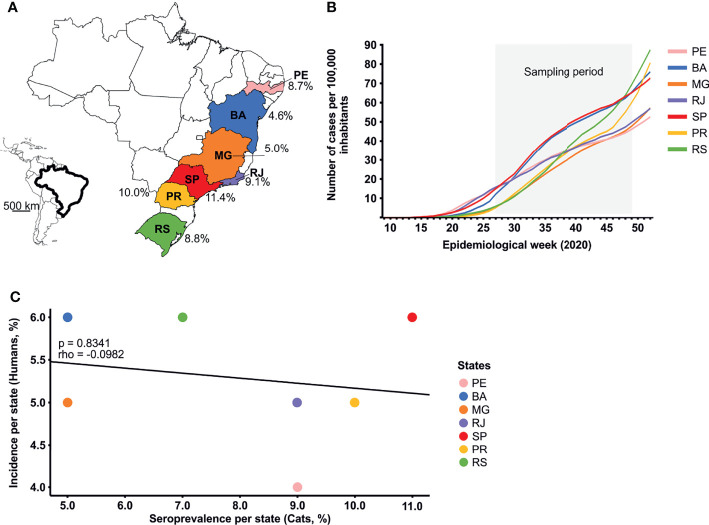
Sampling sites and COVID-19 incidence. **(A)** SARS-CoV-2 seroprevalence in cats in different states in Brazil. **(B)** Weekly SARS-CoV-2 incidence per 100,000 habitants among different states in Brazil. **(C)** Spearman’s rank correlation comparing SARS-CoV-2 seroprevalence in cats and cumulative cases in humans per state by November 2020 in Brazil. BA, Bahia; MG, Minas Gerais; PE, Pernambuco; PR, Paraná; RJ, Rio de Janeiro; RS, Rio Grande do Sul; SP, São Paulo.

To assess the SARS-CoV-2 seroprevalence in cats in Brazil, we tested a total of 547 sera sampled during the second half of 2020 in seven Brazilian states spanning 3000 km longitude. The overall SARS-CoV-2 seroprevalence confirmed by PRNT_90_ was 7.3% (95% CI, 5.3-9.8), ranging from 4.6 (95% CI, 2.2-8.4) to 11.4 (95% CI, 6.7-17.4) in different states of Brazil (**Table 1** and **Figure 2A**). The regional differences in seroprevalence in cats were not statistically significant (chi-square, p=0.4438), despite differences between the COVID-19 incidence in humans in those Brazilian states (**Figure 2B**). Cumulative incidence in humans and cat seroprevalence were not significantly correlated (rho=-0.0982, p=0.8341; **Figure 2C**), although the rates were similar in some Brazilian states (**Supplementary Figure 1**). Whether cats can thus be used as a proxy for human infection remains to be determined using representative samples.

### Testing for Antibodies Against Enzootic Coronaviruses

We tested all 52 pre-pandemic sera and a subset of 68 pandemic sera showing sufficient volumes against FCoV and BCoV, including all 42 samples that were seropositive for SARS-CoV-2.

Only two of those 120 sera contained BCoV-specific neutralizing antibodies (1.7%; 95% CI, 0.2-5.9). To our knowledge, infection of cats with viral strains belonging to the species *Betacoronavirus-1* suggested by our data has not been reported. The only coronavirus comprised in the viral species *Betacoronavirus-1* reported in carnivores so far is Canine Respiratory coronavirus (CrCoV) in dogs (1). Whether and which viruses comprised in the *Betacoronavirus-1* species may infect cats thus requires further investigation. Interestingly, the two cat sera that tested positive for BCoV presented inhibition of 16.7% and 53.6% in the sVNT, including the pre-pandemic serum that would have been considered positive upon using criteria established for testing humans (**Figure 1B**). Our data thus suggest that the sporadic presence of neutralizing antibodies against members of the species Betacoronavirus-1 may elicit cross-reactive antibodies interfering with serological detection of antibodies against the antigenically related betacoronavirus SARS-CoV-2. Our interpretation is supported by a previous study reporting cross-reactivity of immune responses against the endemic HCoV-OC43 and SARS-CoV-2 in humans interfering with antibody detection, particularly when using spike-based serological assays (18).

**Figure 3:**
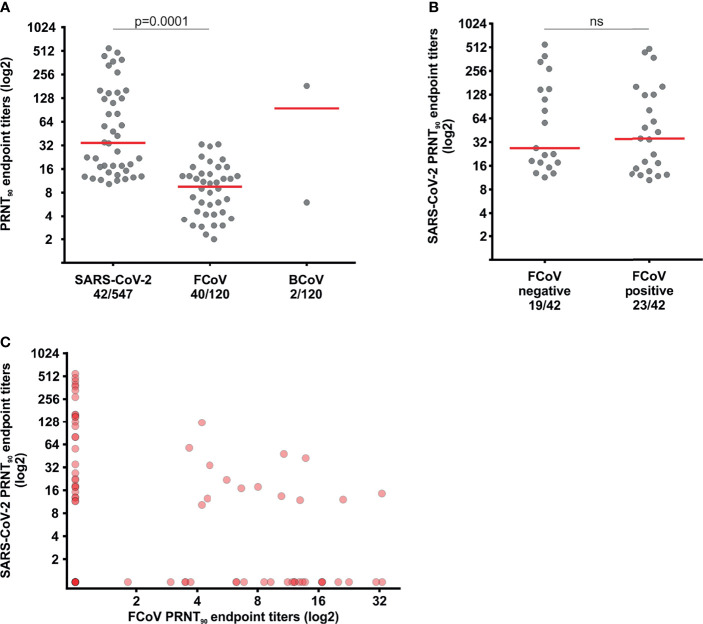
Neutralizing antibodies against SARS-CoV-2 and enzootic coronaviruses. **(A)** Comparison of the reciprocal FCoV, BCoV and SARS-CoV-2 PRNT_90_ endpoint titers in cat sera. **(B)** Comparison of SARS-CoV-2 and FCoV PRNT_90_ endpoint titers. **(C)** Reciprocal SARS-CoV-2 PRNT_90_ endpoint titers among FCoV-positive and -negative sera. Statistical significance was inferred using the Mann-Whitney test in **(A)** and **(B)**. The red bars indicate medians; ns, not statistically significant.

In contrast, FCoV-specific neutralizing antibodies were detected in 33.3% of cats (40/120, 95% CI, 24.9-42.5) (**Figure 3A**). The FCoV seroprevalence found here is comparable with data reported previously from Brazil, whereas seroprevalence rates in cats ranged from 13.2 to 56.2% in different age groups (29, 30). The disparities of seroprevalence by age are because over 70% of FCoV infections occur in animals with less than 1 year of age and FCoV antibody titers decrease with time (31, 32). Cats remain seropositive for prolonged time spans following SARS-CoV-2 and FCoV infection (33, 34), however around 12% of FCoV-infected animals are lifelong carriers and antibody responses among those animals can differ from animals who have cleared FCoV infection (32, 34). Therefore, comparisons between FCoV and SARS-CoV-2 titers over time should be interpreted with caution. Notably, our results are unlikely to be related with vaccination of cats, since the single existing FCoV vaccine is not available in Brazil (32). Moreover, the median PRNT_90_ endpoint titer among seropositive sera was about 3.5x higher for SARS-CoV-2 than for FCoV (**Figure 3A**, p=0.0001), supporting a relatively more recent infection of cats with SARS-CoV-2 in contrast to prior infection with the enzootic FCoV (**Figure 3A**). Neither the SARS-CoV-2 seroprevalence (chi-square, p=0.8863), nor the magnitude of SARS-CoV-2 endpoint titers (Mann-Whitney test, p=0.6492) differed between FCoV-positive and FCoV-negative sera (**Figures 3B, C**), suggesting that pre-existing immunity against enzootic carnivore coronaviruses did not affect subsequent SARS-CoV-2 infection.

## Discussion

We revealed geographically widespread and frequent infection of cats from Brazil with SARS-CoV-2. The few available studies on SARS-CoV-2 epidemiology in cats globally show considerable discrepancy in seroprevalence rates (9, 35–39). Those discrepancies are likely related to different antibody detection techniques and sampling strategies. The highest seroprevalence rates were described upon testing cats from COVID-19 positive households, e.g., 14.8% of cats in Wuhan, China (9) and, 58.8% of cats in France (39). Regardless, the seroprevalence in Brazil was higher than those reported from domestic cats in Europe during early 2020, namely 0.7 to 1.4% in Germany, 0.8% in Croatia and 3.3% in the UK (5, 35–38) and 0.8% found in cat shelters in the Netherlands (40), likely consistent with less intense SARS-CoV-2 circulation in humans during those studies. On the other hand, our data is consistent with seroprevalence rates of 4.2 to 5.8% in Italy (5, 38), 4.2% Germany and 6.4% in Spain (38), likely due to relatively higher SARS-CoV-2 incidence during late 2020 and in those countries. Altogether, those comparisons substantiate the robustness of our data showing a relatively high seroprevalence in Brazilian cats likely facilitated by intense SARS-CoV-2 transmission in Brazil during the study period.

The immune interplay between different coronaviruses in different hosts is poorly understood. In humans, immunity against common cold coronaviruses has been reported to afford cross-protection against subsequent SARS-CoV-2 infection (41). In contrast, some studies have reported a tentative association between prior immunity against common cold coronaviruses and COVID-19 severity, hypothetically either *via* enhancement or *via* insufficient mounting of SARS-CoV-2 specific immune responses (1, 18, 25, 42). We found no evidence that pre-existing immunity against enzootic carnivore coronaviruses protects against or enhances SARS-CoV-2 infection. Our interpretation is supported by previous findings in humans showing the absence of neutralizing activity against SARS-CoV-2 among individuals recently infected with endemic human coronaviruses (43). It is feasible to hypothesize that cross-protection between FCoV and SARS-CoV-2 is unlikely, however, since ADE may be related to antibody concentrations (20, 44) and very few clinically symptomatic SARS-CoV-2 infections have been so far reported in cats (4, 8), it remains unclear whether ADE pottentially impacts the clinical outcome of SARS-CoV-2 infection in cats. Extrapolating from other viruses known for potential ADE such as flaviviruses, it seems likely that the chronology and sequence of prior infections, in addition to inter-individual variation of immune responses may affect the coronaviral immune interplay (45).

Our study was limited by our sampling of animals presenting clinical disease. It is possible that common cat pathogens that were not tested for here are more frequent in our samples than in clinically healthy cats. For instance, those pathogens could have elicited unspecific antibody responses due to acute infections potentially causing polyclonal B-cell stimulation such as observed in humans (46).

In summary, our results substantiate high SARS-CoV-2 seroprevalence in cats from Brazil already in late 2020. It seems plausible that seroprevalence has increased since the time of our study. Genetic characterizations of SARS-CoV-2 strains from cats and humans in a One Health framework to monitor the emergence of potential mutations of concern may allow early detection of cat-associated SARS-CoV-2 variants, and potential preventive control measures such as vaccination of animals (47).

## Data Availability Statement

The raw data supporting the conclusions of this article will be made available by the authors, without undue reservation.

## Ethics Statement

The animal study was reviewed and approved by the Federal University of Bahia’s animal ethics committee under authorization no. 74/2019. Written informed consent for participation was not obtained from the owners because it was not required by the Ethical Committee because the sera used in this study were leftovers of samples sent for medical diagnostics.

## Author Contributions

EO-F, OC, IC, FF, SN, CP, LG-A, VC, AK, RM, WJ, AM-S, CR, CD, KO, CB, EN, LR, RM, FF, NR, CF, and JD conceived, planned and performed the experiments. EO-F and AK carried out the BSL-3 experiments. EO-F, OC, FF, VU, RM, IC, AM-S, and JD planned and carried out the analyses and contributed to the interpretation of the results. EO-F and JD took the lead in writing the manuscript. All authors provided critical feedback, helped shape the research, analysis, and manuscript. All authors contributed to the article and approved the submitted version.

## Conflict of Interest

Authors OC and LR were employed by company Tecsa Laboratorios.

The remaining authors declare that the research was conducted in the absence of any commercial or financial relationships that could be construed as a potential conflict of interest.

## Publisher’s Note

All claims expressed in this article are solely those of the authors and do not necessarily represent those of their affiliated organizations, or those of the publisher, the editors and the reviewers. Any product that may be evaluated in this article, or claim that may be made by its manufacturer, is not guaranteed or endorsed by the publisher.
