# Supplementary figures and images for: Frequent Infection of Cats With SARS-CoV-2 Irrespective of Pre-Existing Enzootic Coronavirus Immunity, Brazil 2020
Source: Front Immunol. 2022 Apr 5;13:857322. doi: 10.3389/fimmu.2022.857322 (PMC9016337; doi:10.3389/fimmu.2022.857322)

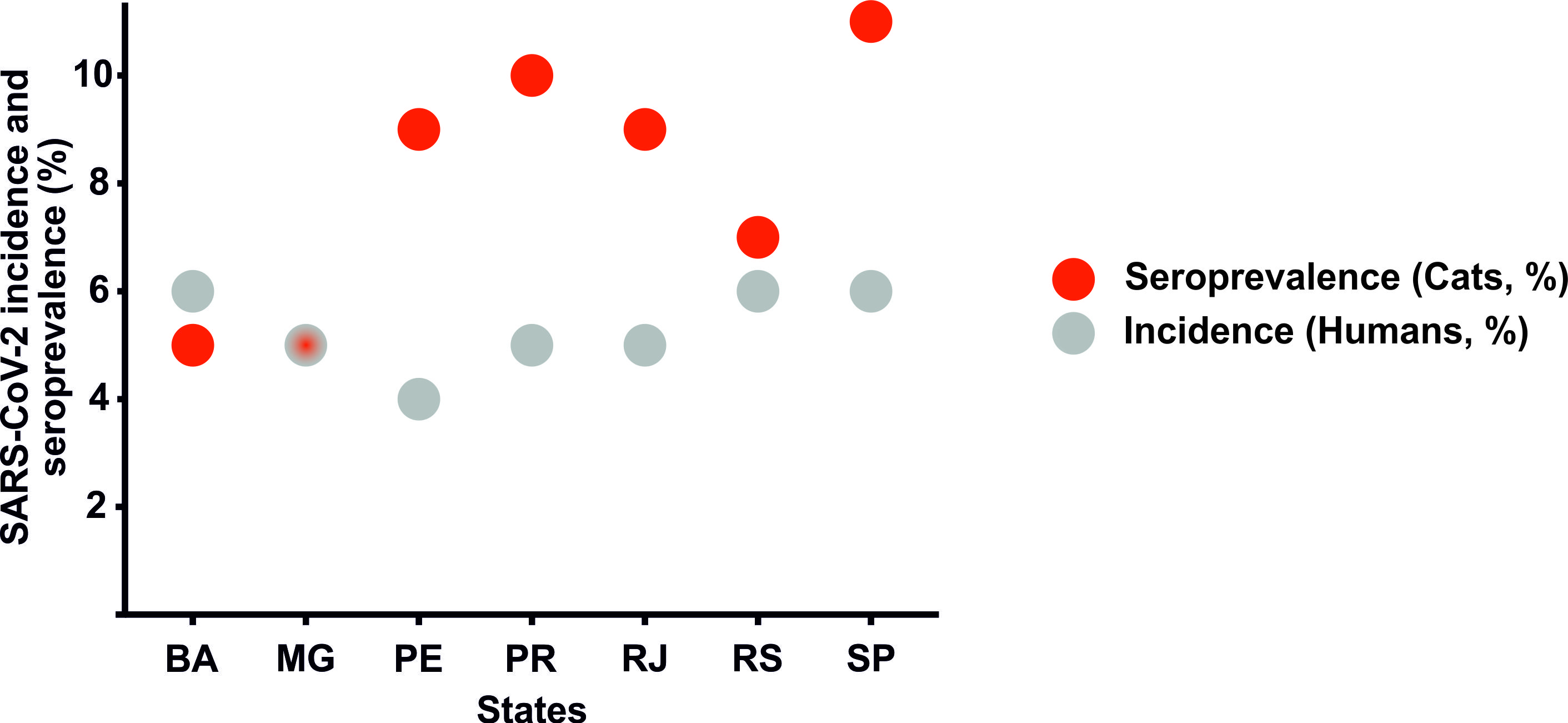

Supplement: Supplementary file 1 [file Image_1.jpeg]
